# In Silico Analysis of Tetrodotoxin Binding in Voltage-Gated Sodium Ion Channels from Toxin-Resistant Animal Lineages

**DOI:** 10.3390/md20110723

**Published:** 2022-11-18

**Authors:** Shana L. Geffeney, Jennie Ann Cordingley, Kenyon Mitchell, Charles T. Hanifin

**Affiliations:** Department of Biology, Utah State University-Uintah Basin, 320 N. Aggie Blvd., Vernal, UT 84078, USA

**Keywords:** marine toxin, voltage-gated ion channels, structure, tetrodotoxin, TTX, toxin resistance, sodium channel, convergent evolution

## Abstract

Multiple animal species have evolved resistance to the neurotoxin tetrodotoxin (TTX) through changes in voltage-gated sodium ion channels (VGSCs). Amino acid substitutions in TTX-resistant lineages appear to be positionally convergent with changes in homologous residues associated with reductions in TTX block. We used homology modeling coupled with docking simulations to test whether positionally convergent substitutions generate functional convergence at the level of TTX–channel interactions. We found little evidence that amino acids at convergent positions generated similar patterns among TTX-resistant animal lineages across several metrics, including number of polar contacts, polar contact position, and estimates of binding energy. Though binding energy values calculated for TTX docking were reduced for some TTX-resistant channels, not all TTX-resistant channels and not all of our analyses returned reduced binding energy values for TTX-resistant channels. Our results do not support a simple model of toxin resistance where a reduced number of bonds between TTX and the channel protein prevents blocking. Rather models that incorporate flexibility and movement of the protein overall may better describe how homologous substitutions in the channel cause changes in TTX block.

## 1. Introduction

Convergent evolution describes the evolution of similar phenotypes in evolutionarily divergent lineages [1,2]. Classic examples include shared body morphologies in fish and aquatic mammals or the evolution of shared ecotypes in placental and marsupial mammals [3,4]. The process can also describe molecular evolution, in which adaptive changes in orthologous genes or homologous positions in proteins underlie the evolution of similar phenotypes [5,6]. At the level of the protein, convergent evolution can inform our understanding of important structure–function relationships as well as functional constraints that may limit the number of “solutions” to selection [7]. Specifically, convergent evolutionary outcomes may suggest that the complex mix of selection and functional constraints mediating protein evolution may be similar across lineages [6].

A striking example of apparent convergent protein evolution is the evolution of TTX-resistant voltage-gated sodium ion channels (VGSCs) in salamanders, snakes, and octopuses that are exposed to the neurotoxin tetrodotoxin (TTX) [8,9,10,11,12]. Salamanders in the genus *Taricha* possess TTX, which they use as a chemical defense [13]. Similarly, blue-ringed octopuses (Genus *Hapalochlaena*) possess high levels of TTX, which they use for both defense and as a venom [14,15,16,17]. Garter snakes in the genus *Thamnophis* consume TTX-bearing *Taricha* newts and are engaged in complex arms races across the range of sympatry between newt and snake populations in western North America [18,19,20,21]. All of these species have evolved organismal resistance to TTX through adaptive substitutions in VGSCs, the proteins that are responsible for the initiation and propagation of action potentials in most metazoans [22]. Although organismal resistance to TTX requires adaptation in multiple VGSC paralogs (e.g., both muscle- and nerve-specific proteins), a key step in the TTX-resistant phenotype appears to be the evolution of TTX resistance in the primary channel expressed in skeletal muscle (Na_V_1.4; newts and snakes) or in the primary TTX-sensitive ortholog in neurons (Na_V_1; *Hapalochlaena* octopuses).

**Figure 1:**
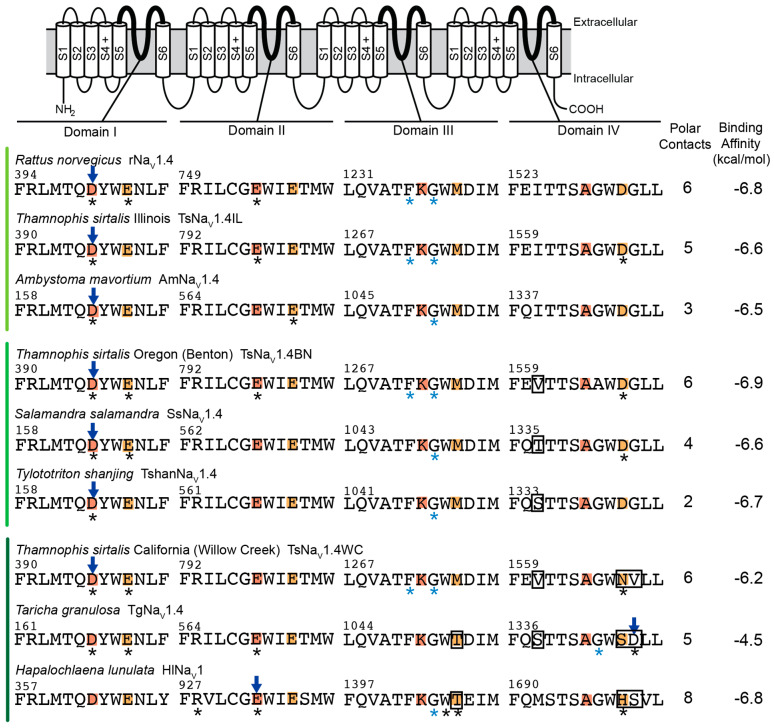
Summary of results for TTX docking in homology models of both TTX-resistant and TTX-sensitive voltage-gated sodium ion channels (VGSCs) using AutoDock Vina rigid channel analysis (VINA-R). Species names and channel protein names are listed above. Amino acid sequences from all four domains of the voltage-gated sodium ion channel that form the TTX binding site are aligned above. The channels are grouped by levels of resistance to TTX blocking: light green—low, medium green—moderate and dark green—“super-resistant” channels, along with a tetrodotoxic octopus *Hapalochlaena lunulata*. The selectivity filter (DEKA) and outer negatively charged ring (EEMD) pore motifs are shaded orange and yellow, respectively. Amino acid positions that form polar contacts with TTX are labeled with asterisks: black for contacts with atoms in sidechains and blue for contacts with atoms in the backbone. The amino acid position that forms a polar contact with the guanidine group on TTX is labeled with an arrow. The number of polar contacts identified by PyMOL between the channels and TTX in the pose with the lowest binding energy (mode 1 conformation) are listed. The binding affinity values, calculated using AutoDock Vina to dock TTX in the VGSCs, are listed. Amino acid substitutions at homologous positions in TTX-resistant channels are labeled with a box. A diagram of a typical VGSC is included to identify the regions in each of the four protein domains that together form the outer pore of the channel.

Convergent evolution in *Taricha*, *Thamnophis*, and *Hapalochlaena* extends beyond the gene level to include within-protein patterns of convergence. The ion-conducting pore of metazoan voltage-gated sodium channels is formed from four homologous protein domains that comprise the VGSC alpha subunit [23]. The outer pore of the channel is composed of amino acids from each of these domains and contains the TTX binding site, the ion selectivity filter, and other structures that modulate channel activity and ion conductance [24,25,26,27,28,29,30,31,32]. Two highly conserved canonical amino acid motifs, DEKA and EEMD (sometimes EEID in invertebrates), form two rings of mostly negatively charged amino acids at the opening of the channel pore [30,33]. Amino acids in the innermost ring (DEKA) form the selectivity filter and interact directly with TTX [26,27,29,30]. Similarly, amino acids in the EEMD motif interact with TTX and modulate ion permeability [30,34]. Substitutions in all domains of the VGSC can render the channel TTX-insensitive but changes associated with resistance in Na_V_1.4 from *Taricha* and *Thamnophis* as well as Na_V_1 from *Hapalochlaena* are localized in domains III and IV [9,10,12]. Strikingly, *Taricha* and *Thamnophis* Na_V_1.4 as well as *Hapalochlaena* Na_V_1 all possess substitutions in the domain IV aspartic acid residue (D) of the EEMD motif as well as substitutions at an adjacent downstream residue (Figure 1). Channels from *Taricha* newts and *Hapalochlaena* octopuses share another amino acid substitution in the EEMD motif in which a threonine replaces the methionine (M) in domain III. Furthermore, *Taricha* and *Thamnophis* channels both possess substitutions in homologous isoleucine residues near the domain IV alanine residue (A) of the DEKA selectivity filter (Figure 1). Together, *Taricha* and *Thamnophis* Na_V_1.4 channels with these substitutions are extremely resistant to TTX block and require TTX concentrations in the 10–100 µM range to block channel function, more than 1000 times the concentrations that block TTX-sensitive Na_V_1.4 channels from salamanders and snakes [9,10].

A general explanation for the convergent patterns of amino acid substitutions seen in newt, snake, and octopus sodium channels is that the “solution space” to evolve a novel phenotype such as TTX resistance is limited by function [1,2,6]. Specifically, only a limited subset of substitutions will result in a protein that is still functional while at the same time include the novel phenotype. Voltage-gated sodium channel genes have been shown to experience strong purifying selection through most of the protein-coding portion of the genes [8]. Decades of work to understand the relationship between structure and function of these proteins have confirmed that complex interactions throughout the protein modulate fundamental properties, such as activation and inactivation, as well as toxin binding [35,36,37]. Furthermore, TTX binds specifically to a limited set of amino acid residues in VGSCs. Taken together, these elements suggest that the “solution space” to evolve TTX resistance may be limited to a small number of sites within the protein and that convergence across lineages results from these constraints.

Current models of TTX interaction with VGSCs suggest that residues in the DEKA and EEMD motifs along with a limited number of other highly conserved residues, such as an aromatic amino acid positioned one residue downstream of the domain I aspartic acid (D) in the DEKA motif, modulate TTX binding [30,34,38,39,40]. Cryogenic EM studies of TTX bound to both invertebrate (Na_V_PaS) and vertebrate (hNa_V_1.7) VGSCs demonstrate that TTX forms polar contacts with amino acids in both of the negatively charged rings (DEKA and EEMD) as well as backbone amides of residues in the domain III portion of the channel pore [41,42]. These studies identified that an amino acid substitution at a conserved position in the pore shifts the number and position of polar contacts (e.g., hydrogen bonds and/or salt bridges) between TTX and amino acids in the channel. Models of TTX binding based on electrophysiological studies incorporate changes in the number and position of polar contacts between TTX and VGSCs to explain how amino acid substitutions in VGSCs alter toxin block [34,39,40]. The goal of our work was to establish whether amino acid substitutions that are linked to reduced toxin block in snake and newt channels also change the number and position of polar contacts between TTX and VGSCs, and if those changes converge on the same solution to alter how TTX binds in the pore.

We modelled the interaction of TTX with a subset of TTX-resistant and non-resistant VGSCs from snakes, newts, and the blue-ringed octopus to directly test the hypothesis that the extreme resistance measured in some of these channels results from convergent modification of toxin binding. Specifically, we used homology modeling coupled with docking analysis (AutoDock Vina (VINA) and GNINA) to model TTX bound to these channels to estimate the number and position of polar contacts between TTX and the channel proteins. Our models incorporated both rigid and flexible amino acid sidechains (VINA-R, GNINA-R and GNINA-F) and allowed us to test the simple hypothesis that TTX resistance in these channels results from a convergent and predictable change in polar contacts between TTX and resistant VGSCs. Because we used TTX-resistant and non-resistant channels from two separate lineages for our analysis (e.g., different salamander species and different populations of the same snake species), we were able to examine within-lineage shifts in the formation of polar contacts to identify convergent patterns among lineages.

## 2. Results and Discussion

### 2.1. Overall Results

Results from all three of our analyses failed to provide strong evidence to support the simple hypothesis that adaptive resistance results from convergent reductions or shifts in polar contacts within and among lineages. In fact, two approaches that maintained rigid amino acid sidechains (VINA-R and GNINA-R) suggested that polar contacts increased as correlates of increased TTX resistance in snakes and salamanders. The inclusion of flexible sidechains coupled with a more computationally intensive analyses (GNINA-F) did suggest that total number of polar contacts in super-TTX-resistant snake and salamander channels were reduced relative to their TTX-sensitive relatives, but polar contact loss was not convergent and did not occur at the same amino acid positions in these lineages. Data from estimates of binding energies within and among TTX-resistant lineages also failed to generate any pattern that was consistent with functional convergence associated with homologous substitutions.

### 2.2. Polar Contact Number

**Figure 2:**
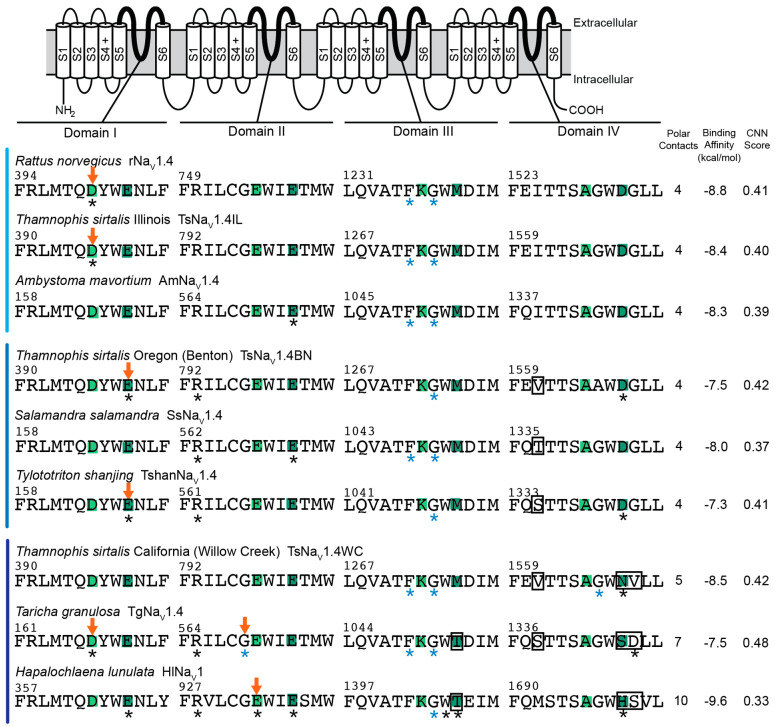
Summary of results for TTX docking in homology models of both TTX-resistant and TTX-sensitive voltage-gated sodium ion channels (VGSCs) using GNINA rigid channel analysis (GNINA-R). Species names and channel protein names are listed above. Amino acid sequences from all four domains of the voltage-gated sodium ion channel that form the TTX binding site are aligned above. The channels are grouped by levels of resistance to TTX block: light blue—low, medium blue—moderate, and dark blue—“super-resistant” channels, along with a tetrodotoxic octopus *Hapalochlaena lunulata*. The selectivity filter (DEKA) and outer negatively charged ring (EEMD) pore motifs are shaded light and dark green, respectively. Amino acid positions that form polar contacts with TTX are labeled with asterisks: black for contacts with atoms in sidechains and blue for contacts with atoms in the backbone. The amino acid positions that form polar contacts with the guanidine group on TTX are labeled with an arrow. The number of polar contacts identified by PyMOL between the channels and TTX in the pose with the lowest binding energy are listed. The binding affinity values and convolutional neural network (CNN) scores, calculated using GNINA to dock TTX in the VGSCs, are listed. Amino acid substitutions at homologous positions in TTX-resistant channels are labeled with a box. A diagram of a typical VGSC is included to identify the regions in each of the four protein domains that together form the outer pore of the channel.

**Figure 3:**
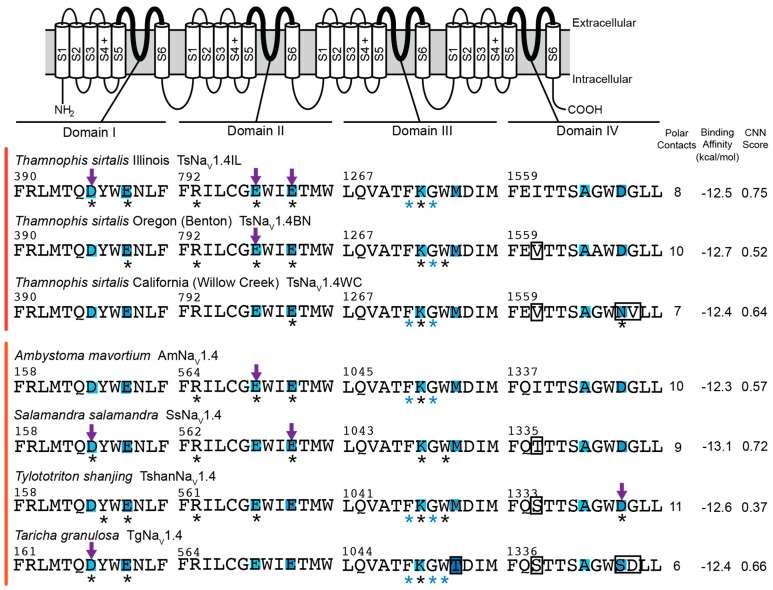
Summary of results for TTX docking in homology models of both TTX-resistant and TTX-sensitive voltage-gated sodium ion channels (VGSCs) using GNINA flexible sidechain analysis (GNINA-F). The search volume for docking was set using TTX bound in the TTX-sensitive channel for each lineage (snake TsNa_V_1.4IL and salamander AmNa_V_1.4) using GNINA rigid docking analysis (GNINA-R). Species names and channel protein names are listed above. Amino acid sequences from all four domains of the voltage-gated sodium ion channel that form the TTX binding site are aligned above. The channels are grouped by animal lineage; red (*Thamnophis sirtalis*) garter snake and orange salamander. The selectivity filter (DEKA) and outer negatively charged ring (EEMD) pore motifs are shaded light and dark blue, respectively. Amino acid positions that form polar contacts with TTX are labeled with asterisks: black for contacts with atoms in sidechains and blue for contacts with atoms in the backbone. The amino acid positions that form polar contacts with the guanidine group on TTX are labeled with arrows. The number of polar contacts identified by PyMOL between the channels and TTX in the pose with the lowest binding energy are listed. The binding affinity values and convolutional neural networks (CNN) scores, calculated using GNINA to dock TTX in the VGSCs, are listed. Amino acid substitutions at homologous positions in TTX-resistant channels are labeled with a box. A diagram of a typical VGSC is included to identify the regions in each of the four protein domains that together form the outer pore of the channel.

**Figure 4:**
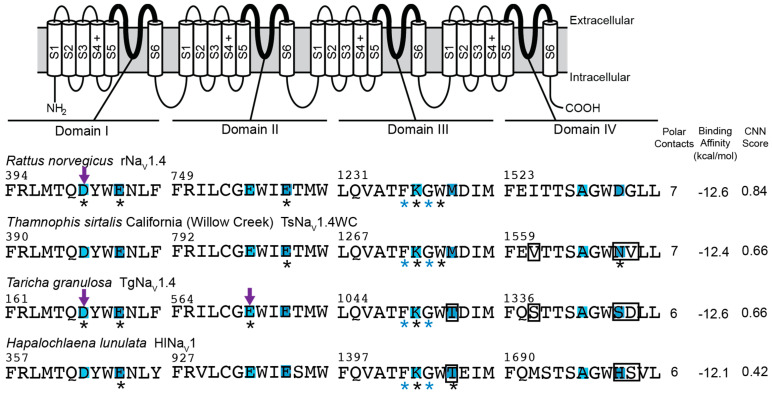
Summary of results for TTX docking in homology models of both TTX-resistant and TTX-sensitive voltage-gated sodium ion channels (VGSCs) using GNINA flexible sidechain analysis (GNINA-F). The search volume for docking was set using TTX bound in each channel using GNINA rigid docking analysis (GNINA-R). Species names and channel protein names are listed above. Amino acid sequences from all four domains of the voltage-gated sodium ion channel that form the TTX binding site are aligned above. The selectivity filter (DEKA) and outer negatively charged ring (EEMD) pore motifs are shaded light and dark blue, respectively. Amino acid positions that form polar contacts with TTX are labeled with asterisks, black for contacts with atoms in sidechains and blue for contacts with atoms in the backbone. The amino acid positions that form polar contacts with the guanidine group on TTX are labeled with arrows. The number of polar contacts identified by PyMOL between the channels and TTX in the pose with the lowest binding energy are listed. The binding affinity values and convolutional neural networks (CNN) scores, calculated using GNINA to dock TTX in the VGSCs, are listed. Amino acid substitutions at homologous positions in TTX-resistant channels are labeled with a box. A diagram of a typical VGSC is included to identify the regions in each of the four protein domains that together form the outer pore of the channel.

Analyses of TTX-sensitive channels provide a baseline estimate for the number of polar contacts between TTX and channels. However, we observed dramatic variation across models and lineages for TTX-sensitive channels (Figure 1, Figure 2, Figure 3 and Figure 4). For example, VINA-R predicted a docked position for TTX that forms six polar contacts with the TTX-sensitive mammalian channel (rNa_V_1.4), while the GNINA-R model forms four and the GNINA-F model forms seven (Figure 1, Figure 2 and Figure 4). Similarly, the TTX-sensitive snake channel (TsNa_V_1.4IL) forms five (VINA-R), four (GNINA-R), and eight (GNINA-F) polar contacts with docked TTX (Figure 1, Figure 2 and Figure 3). Interestingly, the TTX-sensitive salamander channel (AmNa_V_1.4) was predicted to form fewer contacts with models that included rigid sidechains: three (VINA-R) and four (GNINA-R), but an increased number (10) for the model that incorporated flexible sidechains (GNINA-F).

Consistent with our simple model of convergent evolution of TTX resistance among lineages, we would expect to see parallel reductions in polar contacts in our known “super-resistant” channels (snake TsNa_V_1.4WC and salamander TgNa_V_1.4), but our results do not support this outcome. In fact, models that did not incorporate flexible amino acid sidechains (VINA-R and GNINA-R) actually predicted greater numbers of polar contacts between TTX and the TTX binding site (Figure 1 and Figure 2). For example, the VINA-R model forms six polar contacts with TsNa_V_1.4WC compared to five with the sensitive TsNa_V_1.4IL, and the same analysis estimated five polar contacts with TgNa_V_1.4 and three with the sensitive salamander channel AmNa_V_1.4 (Figure 1). Models that incorporated flexible sidechains (GNINA-F) did provide limited evidence that polar contact formation was reduced in “super-resistant” snake and newt channels (Figure 3). This analysis predicted bound TTX positions that form 8 and 10 polar contacts with sensitive snake (TsNa_V_1.4IL) and sensitive salamander (AmNa_V_1.4), respectively, and a reduced number for “super-resistant” snake (7; TsNa_V_1.4WC) and salamander (6; TgNa_V_1.4). Interestingly, this downward trend results from increases in estimated polar contacts for the sensitive channels in both lineages. Finally, the octopus channel (HlNa_V_1), which shares similar substitutions with both TsNa_V_1.4WC and TgNa_V_1.4, was predicted to form more polar contacts with TTX in the fixed sidechain analyses (8, VINA-R and 10, GNINA-R) and fewer in the flexible sidechain analysis (6, GNINA-F; Figure 1, Figure 2 and Figure 4).

Polar contact predictions for intermediate TTX-resistant snake and salamander channels (TsNa_V_1.4BN, snake; TshanNa_V_1.4 and SsNa_V_1.4, salamander) provide no support for our hypothesis (Figure 1, Figure 2 and Figure 3). Polar contact estimates for these channels ranged from a low of 2 (TshanNa_V_1.4, VINA-R) to a high of 11 (TshanNa_V_1.4, GNINA-F). Even when compared within lineages and within different models, our intermediate channels did not form intermediate numbers of polar contacts (Figure 1, Figure 2 and Figure 3). In snakes, TsNa_V_1.4BN had either the same or a higher number of polar contacts compared to the sensitive TsNa_V_1.4IL for all analyses, and the same general pattern was true for both of the intermediate salamander channels (Figure 1, Figure 2 and Figure 3), though TshanNa_V_1.4 (VINA-R) and SsNa_V_1.4 (GNINA-F) form one less in individual analyses.

### 2.3. Polar Contact Position Shifts

Our results provide little evidence that convergent changes in polar contact position underlie the evolution of adaptive resistance to TTX in snake, salamander, and octopus channels. Although results from our analyses provide some support that TTX resistance is associated with shifts in the position of polar contacts, they do not generate a clear pattern of polar contact shifts among “super-resistant” channels that suggest the changes are similar across lineages (Figure 1, Figure 2, Figure 3 and Figure 4). For snakes (TsNa_V_1.4IL, TsNa_V_1.4BN, and TsNa_V_1.4WC), data from the GNINA analyses (GNINA-R and F) suggest that resistance in TsNa_V_1.4WC results from a loss of polar contacts with amino acids in domain I and II and an increase in polar contacts with amino acids in domain III and IV, but these shifts are not supported by VINA-R and we see no equivalent pattern in salamanders (Figure 1, Figure 2, Figure 3 and Figure 4).

Our results predict that the TTX-sensitive snake (TsNa_V_1.4IL) and mammal (rNa_V_1.4) channels form polar contacts between TTX and amino acids in the selectivity filter as well as backbone atoms from residues in domain III (Figure 1, Figure 2 and Figure 3) and are consistent with current understanding of the interaction between TTX and TTX-sensitive VGSCs [41,42]. Additionally, data from the VINA-R and GNINA-F analyses predict that TTX-sensitive snake (TsNa_V_1.4IL) and rat (rNaV1.4) channels form polar contacts with TTX at amino acid position in the outer negatively charged ring (Figure 1, Figure 3 and Figure 4). These results are also consistent with general models of TTX-channel interactions [41,42]. Notably, all of the TTX-sensitive snake channel docking models from all analyses form polar contacts with TTX and an aspartic acid in the domain I portion of the selectivity filter (D396, Figure 1, Figure 2 and Figure 3). Additionally, the majority (two of three) of the channel docking models form a polar contact with TTX at the glutamic acid in the domain II portion of the selectivity filter (E798, Figure 1, Figure 2 and Figure 3). Polar contact formation between TTX and the two negatively charged amino acids (D396 and E798) in the selectivity filter may be an important component of channel block, because the selectivity filter is the narrowest portion of the open pore and these negatively charged amino acids may coordinate sodium ion movement through the pore [41,43].

**Figure 5:**
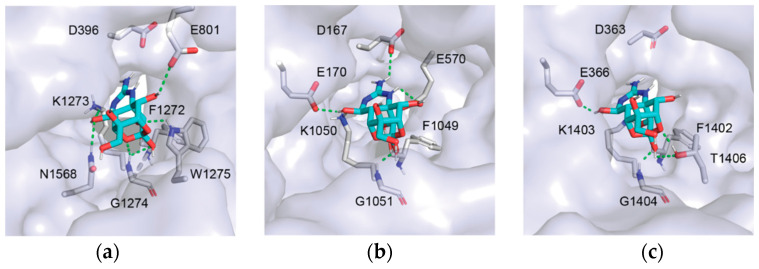
Structure of TTX docked in skeletal muscle sodium channel proteins Na_V_1.4 from a “super-resistant” garter snake, *Thamnophis sirtalis* (**a**) and salamander *Taricha granulosa* (**b**), as well as a neuronal sodium channel protein from the tetrodotoxic Greater Blue-Ringed Octopus *Hapalochlaena lunulata* Na_V_1 (**c**). The overall channel protein structure is shown as a surface view. Polar contacts between TTX and amino acids in the channel protein are shown as green dotted lines. Amino acids that form polar contacts with TTX are shown in each channel. The guanidine group on TTX does not form a polar contact with amino acids in the snake channel TsNa_V_1.4WC (**a**) or the octopus channel HlNa_V_1 (**c**). In contrast, the guanidine group on TTX forms polar contacts with two amino acids in the selectivity filter of the salamander channel TgNa_V_1.4 (**b**) in domain I at D167 and domain II at E570.

Data from TTX-resistant snake channels suggest that amino acid substitutions linked to reduced TTX block alter the position of polar contacts between TTX and amino acids in the TTX binding site (Figure 2, Figure 3 and Figure 4). Data from the GNINA-F analysis predicted that the TTX-sensitive snake channel (TsNa_V_1.4IL) forms polar contacts between TTX and both negatively charged amino acids in the selectivity filter (D396 and E798, Figure 3). In the moderately resistant snake (TsNa_V_1.4BN) channel, polar contacts are altered and the channel only forms a contact with one of these two amino acids, the glutamic acid in domain II (E798, Figure 3). The polar contacts are altered again in the “super-resistant” snake channel (TsNa_V_1.4WC) and no polar contacts are formed between TTX and the channel at these two positions (Figure 3, Figure 4 and Figure 5). Instead, TsNa_V_1.4WC forms contacts with amino acids in the outer negatively charged ring in domain II and IV, as well as the same set of amino acids in domain III as other snake channels. Data from the GNINA-R analysis predicted a similar shift in polar contacts (Figure 2). The TTX-sensitive snake channel (TsNa_V_1.4IL) forms a polar contact with TTX at the aspartic acid in the selectivity filter (D396). However, neither the “super-resistant” or moderately resistant snake channels (TsNa_V_1.4WC and TsNa_V_1.4BN) form polar contacts between TTX and the negatively charged amino acids in the selectivity filter.

In contrast, data from the GNINA analyses (GNINA-F and GNINA-R) do not provide a clear a pattern of polar contact shifts for channels in the salamander lineage (Figure 2, Figure 3 and Figure 4). Data from the GNINA-F analysis predicted that all channels in this lineage form contacts with either the negatively charged amino acid in the domain I or domain II portion of the selectivity filter (Figure 3). Additionally, a separate analysis of TTX docking with the GNINA-F tool predicted that the “super-resistant” salamander channel (TgNa_V_1.4) would form polar contacts with TTX at both negatively charged amino acids in the selectivity filter (D167 and E570, Figure 4 and Figure 5). Data from the GNINA-R analysis of channels in the salamander lineage did not predict polar contact shifts away from the negatively charged amino acids in the selectivity filter of the “super-resistant” salamander channel (TgNa_V_1.4, Figure 2). TTX-sensitive channels and moderately resistant salamander channels were not predicted to form polar contacts between TTX and the two negatively charged amino acids in the selectivity filter, and the “super-resistant” salamander channel was predicted to form a polar contact with the aspartic acid in the domain I portion of the selectivity filter (D167, Figure 2).

The blue-ringed octopus (HlNa_V_1) channel shares amino acid substitutions at multiple positions with both “super-resistant” snake and salamander channels. Data from the GNINA-F analysis predicted that these substitutions would change the pattern of polar contacts in a similar way to the “super-resistant” snake channel; however, data from other analyses did not follow this pattern (Figure 1, Figure 2 and Figure 4). The toxin in the position predicted by GNINA-F analysis did not form polar contacts with the octopus channel (HlNa_V_1) at either of the two negatively charged amino acids in the selectivity filter (D363 and E933, Figure 4 and Figure 5). Instead, the channel formed polar contacts with TTX at two amino acids in the outer negatively charged ring, in domain I (E366) and domain III (T1406). In contrast, data from the rigid channel analysis (VINA-R and GNINA-R) predicted that the octopus channel would form a polar contact with the glutamic acid in the domain II position of the selectivity filter (E933, Figure 1 and Figure 2).

The lack of convergent patterns in our data could be the result of fundamentally different structure–function relationships in snake, salamander, and octopus channels. For example, TTX–channel interactions are governed by complex state-dependent factors (e.g., use-dependent block) that are associated with conformational changes as the channel opens. Differences in key residues across snake, salamander, and octopus channels might also modulate these conformational changes and modify the formation of polar contacts between TTX and channels. However, little is known about these processes in non-mammalian channels and our data cannot address this directly. Though our data may not fully capture alterations in channel conformation, data from docking TTX in the TTX-sensitive mammalian channel (rNaV1.4) suggest that our analyses capture some of the important components of channel block (Figure 1, Figure 2 and Figure 4). The data from all three analyses broadly agree with current understanding of the interaction between TTX and TTX-sensitive VGSCs [41,42]. In all of our data, TTX binding with rNa_V_1.4 is coordinated across the pore with the guanidine group on TTX forming a polar contact with the aspartic acid in the domain I portion of the selectivity filter (D400) and other portions of the toxin forming polar contacts with backbone amines in domain III (F1236 and G1238). Additionally, in the VINA-R and GNINA-F analyses, rNaV1.4 forms polar contacts with TTX at amino acid positions in the outer negatively charged ring (E403, VINA-R and E403 and E758, GNINA-F). Taken together, these results suggest that our models are generating appropriate interactions between TTX and VGSCs and could capture shared patterns of polar contact shifts between the snake and salamander lineages.

### 2.4. Binding Affinity and CNN Scoring of TTX Poses

The VINA and GNINA tools use different mechanisms for assessing ligand-channel interactions including ligand binding affinity scores. The VINA tool uses an empirical scoring function based on structural data to calculate the binding affinity of ligands docked in receptors [44]. The GNINA tool uses a form of machine learning to compare and score ligand poses [45]. The GNINA tool calculates a binding affinity score for docked ligands along with a score that evaluates the pose of the ligand, the convolutional neural network (CNN) score. Binding affinity scores from the VINA-R tool used in this analysis suggest that TTX does not bind as well to “super-resistant” snake and salamander channels (Figure 1); however, none of the binding affinity scores from the GNINA tool (GNINA-R and GNINA-F) distinguish between the channels in this analysis (Figure 2, Figure 3 and Figure 4). Instead, the CNN scores for the docked ligands from the GNINA analysis provide some evidence that the flexible sidechain analysis (GNINA-F) may provide better predictions of the position of TTX bound in the channels.

Comparisons of binding energy values based on binding affinity predictions from the VINA tool failed to differentiate between moderate and TTX-sensitive channels, but for known “super-resistant” channels (salamander TgNa_V_1.4 and snake TsNa_V_1.4), this value was a good predictor of TTX resistance (Figure 1). Estimated binding energy values for rat (rNa_V_1.4) and TTX-sensitive snake (TsNa_V_1.4IL) channel were similar −6.8 kcal/mol and −6.6 kcal/mol, respectively (Figure 1). The binding energy estimate for the moderately resistant snake channel (TsNa_V_1.4BN) was slightly more negative than either of these estimates at −6.9 kcal/mol. We estimated reduced binding affinity (−6.2 kcal/mol) for the “super-resistant” snake channel (TsNa_V_1.4WC). Binding energy values for TTX-sensitive and moderately resistant salamander channels were similar to the binding affinity estimates for TTX-sensitive snake channels: −6.5 to −6.7 kcal/mol. By contrast, the binding energy for the “super-resistant” salamander channel (TgNa_V_1.4) was severely reduced at −4.5 kcal/mol. Finally, we estimated a binding energy for the octopus channel (HlNa_V_1) of −6.8 kcal/mol. This value is comparable to the value estimated for the rat channel. These results suggest that the two homologous amino acid substitutions in domain IV that snakes, newts, and octopuses share do not change the interaction of TTX with the channels in the same way. For the “super-resistant” snake (TsNa_V_1.4WC) and salamander (TgNa_V_1.4) channels, we estimated reduced binding affinity between TTX and the channels. However, though we identified changes in the pattern of polar contacts between the octopus channel and TTX compared to other channels, this pattern did not reduce the binding affinity values we calculated for the interaction (Figure 1).

In contrast, none of the binding affinity values predicted by the GNINA tool were good predictors of TTX resistance (Figure 2, Figure 3 and Figure 4). The binding affinity estimates from the rigid channel analysis (GNINA-R) for channels in the snake lineage were all within 1 kcal/mol of each other and the “super-resistant” snake channel had the lowest binding energy estimate of −8.5 kcal/mol. The binding affinity estimates for channels in the salamander lineage were also within 1 kcal/mol and, though the “super-resistant” salamander channel had a more positive value for binding affinity than the TTX-sensitive salamander channel, it was not the most positive value calculated for the lineage. Finally, the octopus channel had the lowest binding affinity estimates of all the channels analyzed.

The binding affinity estimates from the GNINA analysis with flexible sidechains (GNINA-F) were lower for all channels compared to the values estimated using rigid channel GNINA analysis (Figure 2, Figure 3 and Figure 4). The range of values for all channels in the GNINA-F analysis was 1 kcal/mol overall; an intermediate resistance salamander channel had the lowest binding affinity estimate (SsNaV1.4, −13.1 kcal/mol) and the blue ringed octopus had the most positive binding affinity estimate (HlNaV1, −12.1 kcal/mol). There was no clear trend in these values that predicted known TTX resistance for the channels.

Docking analysis with the GNINA tool provides another score of the ligand pose, the convolutional neural network (CNN) score, which evaluates the three-dimensional representation of the ligand poses. The GNINA tool provides CNN score values ranging from 0 to 1, with a score of 1 indicating a perfect ligand pose [45]. We used CNN scores from both rigid channel and flexible sidechain GNINA analyses to evaluate poses with the lowest binding affinity values (Figure 2, Figure 3 and Figure 4). The CNN scores for flexible sidechain docking (GNINA-F) were consistently higher than the CNN scores for rigid channel docking (GNINA-R). The rat channel (rNaV1.4) had the largest score value increase, from 0.41 to 0.84, though the values for one salamander channel decreased (TshanNaV1.4, from 0.41 to 0.37). This overall increase in CNN score values for flexible sidechain docking suggests that flexible docking using the GNINA tool (GNINA-F) may provide better pose estimates for TTX binding to VGSCs than rigid channel docking (GNINA-R).

## 3. Methods

### 3.1. Homology Modeling and Sampling

We used the Swiss–Model server homology modeling pipeline for protein homology modeling [46]. The structure of the human skeletal-muscle voltage-gated sodium ion channel (hNa_V_1.4) was used as a template, against which all amino acid sequences were modeled (6AGF, [43]). We chose this template for two reasons, the high resolution of the selectivity filter in this channel structure (up to 2.8 Å) along with the fact that this structure captures the open-channel conformation of the pore [43]. Because TTX block increases with repeated channel opening, it is likely that TTX binds to and blocks the open conformation of VGSCs [47]. Thus, we analyzed TTX docking in models of the pore in the open-channel conformation.

We analyzed a total of eight VGSCs that included seven vertebrate Na_V_1.4 sequences and a single invertebrate sequence (Na_V_1). We limited our vertebrate sequences to Na_V_1.4 sequences to avoid VGSCs that are TTX insensitive because of selection for function rather TTX resistance (e.g., mammalian cardiac muscle channels; Na_V_1.5). Our sampling included TTX-sensitive channels from a mammal species (rat *Rattus norvegicus*; rNa_V_1.4; P15390), a salamander species (*Ambystoma mavortium*; AmNa_V_1.4; AIX03051), and a *Thamnophis sirtalis* snake population (Illinois; TsNa_V_1.4IL; AYG96483). We also included Na_V_1.4 channels from two salamander species (*Salamandra salamandra* and *Tylototriton shanjing*) and a single population of *Thamnophis sirtalis* from Oregon (Benton) that possess moderate levels of TTX resistance: SsNa_V_1.4 (AIX03050), TshanNa_V_1.4 (AIX03049), and TsNa_V_1.4BN (AAW68222). The moderately resistant channels are blocked by TTX concentrations in the 50–100 nM range [9,10]. *Salamandra* and *Tylototriton* are related to *Taricha* and previous work suggests that they represent evolutionary links between a TTX-sensitive ancestor of all newts and highly TTX-resistant TTX-bearing salamanders such as *Taricha* newts [9]. Finally, we modelled “super-resistant” Na_V_1.4 channels from a salamander species, *Taricha granulosa* (TgNa_V_1.4; AIX03043), a population of highly resistant *Thamnophis sirtalis* from California (Willow Creek; TsNa_V_1.4WC; AAW68224) as well as the primary channel expressed in neurons of all invertebrates (Na_V_1) from the Greater Blue-Ringed Octopus *Hapalochlaena lunulata* (HlNa_V_1; QPI69428). Amino acid sequences were taken from either Genbank or UniprotKB. We used a mix of full coding sequence as well as partial coding sequence for our analysis. Only a partial coding sequence was available for the salamander species we included in our study. To increase the accuracy of our modeling of the channel pore, we used partial sequences that included 27 amino acids upstream of the start of the fifth transmembrane helix in domain I through 10 amino acids beyond the end of the sixth transmembrane helix in domain IV.

### 3.2. TTX Docking

We used two different tools (AutoDock Vina (version 1.2.3, https://vina.scripps.edu/, accessed on 15 January 2022) and GNINA (version 1.0.1, https://github.com/gnina/gnina, accessed on 15 September 2022)) and three different analyses (fixed versus flexible amino acid sidechains, see below) to examine TTX docking for all channels. [44,45,48]. We downloaded the structure of TTX from Protein Data Bank (9SR, [41]) and prepared the TTX ligand for docking. We converted the TTX structure data file (9SR) to a PDB file format using Open Babel software [49] to upload it to AutoDock Tools. We used AutoDock Tools to add all hydrogens as well as all charges to TTX and channels to prepare the homology models and TTX for docking analysis with AutoDock Vina and GNINA.

We used a search volume of 8 Å × 8 Å × 10 Å centered between the selectivity filter and the outer negatively charged ring in the pore of all homology models to dock TTX in fixed-position channel models (AutoDock Vina (VINA-R) and GNINA (GNINA-R)). We used the “autobox_ligand” feature of GNINA (GNINA-F) to define the search volume using docked TTX pdb files from GNINA outputted by the fixed-position channel analysis (GNINA-R) to dock TTX into channel models with flexible sidechains. We used the “flexdist” and “flexdist_ligand” features of GNINA to render all the sidechains within 4 Å of the search volume flexible during docking. All of these analyses yielded the nine best binding poses of TTX (modes) for each homology model ranked by binding affinity score. We used the pose with the lowest energy score for structure analysis as well as for our estimate of binding energy for among channel comparisons.

### 3.3. Structure Analysis

We used PyMOL-pdb viewer (version 2.5.2, https://pymol.org/2/, accessed on 20 August 2021) to visualize TTX docked in the channels [50]. We used PyMOL (version 2.5.2, https://pymol.org/2/, accessed on 20 August 2021) to identify polar contacts between TTX, in the pose with the lowest energy score, and our homology models. PyMOL emulates the DSSP secondary structure assignment algorithm to determine whether atoms form polar contacts [51].

## 4. Conclusions

The results described here suggest that homologous substitutions in different lineages of TTX-resistant vertebrates do not act in a convergent fashion. None of the metrics we estimated, including the number and position of polar contacts, showed evidence of functional convergence in snakes, salamanders, or the TTX-bearing Greater Blue-Ringed Octopus. The lack of support for our hypothesis could result from model-related errors, if, for example, our models lack the resolution to differentiate between closely related channels, or it could result from structural differences in channels that change the effects of homologous substitutions. However, we saw no evidence from model parameters that either of the tools we used was more or less effective in a given lineage. Instead, we argue that our observed results are more consistent with a protein evolution model that predicts context-dependent effects of amino acid substitutions, as we observed for domain III and IV pore substitutions in VGSCs. These results are congruent with a growing body of evidence that contingency plays a critical role in generating the functional and selective value of novel substitutions [52,53]. Although our ability to draw conclusions about the TTX binding site and the mechanisms underlying resistance in modelled channels is limited, our results suggest that the properties of the whole channel, such as flexibility and shape, may play a role in modulating or mediating TTX resistance.

## Data Availability

Structural data will be archived in Dryad.
